# From Mattering to Mattering More: ‘Goods’ and ‘Bads’ in Ageing and Innovation Policy Discourses

**DOI:** 10.3390/ijerph18147596

**Published:** 2021-07-16

**Authors:** Carla Greubel, Ellen H. M. Moors, Alexander Peine

**Affiliations:** Copernicus Institute of Sustainable Development, Utrecht University, P.O. Box 80115, 3508 TC Utrecht, The Netherlands; E.H.M.Moors@uu.nl (E.H.M.M.); a.peine@uu.nl (A.P.)

**Keywords:** ageing and innovation policy discourse, personas, empirical ethics, goods and bads

## Abstract

This paper provides an empirical ethics analysis of the goods and bads enacted in EU ageing and innovation policy discourses. It revolves around a case study of the persona Maria, developed as part of the EU’s Active and Healthy Ageing Policies. Drawing on Pols’ empirical ethics as a theoretical and methodological approach, we describe the variety of goods (practices/situations to be strived for) and bads (practices/situations to be avoided) that are articulated in Maria’s persona. We analyse how certain ideas about good and bad ageing—those associated with the use of sophisticated technologies—come to matter more in the solutions proposed for Maria and the framing of her unmet needs, while others which were initially seen as relevant and that describe her dreams, fears and interactions, are marginalised. The paper adds to existing studies of ageing and technology by analysing specific practices that render visible how the idea of technology and data sharing as evidently the right path towards futures of (good) ageing, comes to prevail.

## 1. Introduction

The observation that technology is widely positioned as a potential solution to deal with population ageing is neither new nor surprising, however, it remains puzzling how exactly technology has prevailed as the logical, inevitable and thus inherently good way to tackle the alleged problems of care and ageing. In fact, this positioning raises questions about the specific forms of ‘good care’ and ‘good ageing’ that are made possible, and that are enacted with technologies as preferred solutions. 

Sharing these concerns, several scholars [1,2,3,4] have expressed the need and offered an invitation to engage in conversations about “how we want to grow, how we want to age, the world we want to be born in, and the technologies we will find in it” [4] (p. 1). In other words, they invite us to think about the *varieties of goodness* [5] that are possible in relation to care and ageing and take this as a starting point from which to consider technological innovation, rather than vice-versa. The aim is thus not to exclude technologies from these conversations, but to allow them to play a more meaningful role as part of an ongoing co-constitution of health, ageing and technology [6].

This article seeks to contribute to the debate by looking into ageing and innovation policy discourses as one of the arenas that shapes how technology is incorporated into the care and everyday lives of older people. We seek to understand the questions of how the seemingly natural link between the (assumed) needs and values of older people—such as ageing at home—and technology as a means of enabling this—such as fall detection sensors—is established (see also [1]). In other words, how is it that digital innovation is often invoked in European old-age policy discourses as a starting point for good ageing and good care? What other ways of framing good ageing can we see in these discourses? Why do they remain implicit, pushed aside, or left ‘forgotten’ [7,8] while others become explicit and central?

In raising these questions, this paper engages with existing empirical and theoretical reflections on the ageing and innovation discourse coming from Science and Technology Studies (STS) and Age Studies [6,9]. Scholars from both fields demonstrate, for instance, how the dominant rhetoric has drawn on a ‘crisis account of ageing’ for many years [10]. This includes reductionist representations of older people and later life that shape the design and use of technologies [11,12,13], and consequently also co-constitute what ageing means, how it is experienced and what values or ideas about ‘good ageing’ it allows for (or not) [11]. Along the same lines, Neven and Peine [2] have pointed out that the ageing and innovation discourse posits digital technologies as the key to ageing futures in order to legitimise large investments in research and innovation projects. It thus forecloses other discourses on what might be useful and what might not be useful technologies for older people. 

The point that technologies play a crucial role in the creation of what we perceive as good ageing futures has been illustrated in various studies. This body of literature also points out the various ‘bads’—common notions of ageing as a ‘problem’ that needs to be solved—that also play a role in policy and innovation discourses. What is strived for, and what is seen as a problem, hang together. Building on these insights, we study how good ageing is imagined and enacted in policy discourses by paying attention not only to the ‘goods’ but also to the ‘bads’ invoked, and to the various relationships between the ‘goods’ and the ‘bads’.

So far, the specific practices through which technology is made a ‘natural’ and ‘inevitable’ part of future ageing and care, and through which specific ‘goods’ and ‘bads’ are also enacted, has received little attention. This article aims to address this gap. In particular, we consider how a focus on innovation relates to the ideas and practices around ‘good ageing’: (a) Based on empirical insights, what can we say about the practices and processes through which the increasing dominance of technology in ageing is made possible in policy discourses? (b) What role do these practices and processes play (or do not play) in the configuration of the goods and bads that come to matter more in these discourses? 

‘Good ageing’ is thus the object of our study, or, as Willems framed it, *varieties of goodness* [5]. He introduced this concept to the study of care and technology as a way to acknowledge that there is not just one version of good care. Both care practices and care technologies articulate and bring about a variety of visions and descriptions of good care that often interrelate in complex ways. This paper draws on the empirical ethics approach outlined by Pols [14,15,16] for the analysis of, and reflection on these goods. This implies, first, that the study of ‘good ageing’ is a study of material and discursive practices in which various notions of the good are shaped and brought into relation with each other. Rather than defining the ‘good’ by formulating normative criteria, empirical ethicists describe the good by analysing what is strived for in specific practices: “[the] rules and routines people live by, ideals they in- or explicitly try to achieve, machines that push them in certain directions, and values that motivate them” [16] (p. 83). These all entail different notions of what it is good to do. At the same time, they also take part in “co-defining the particular problems that need solving” [16] (p. 84), and they express what is to be avoided, and what we analyse as ‘bads’. When looking into policy discourse documents, ‘goods’ and ‘bads’ can thus be studied in statements about what policy discourse actors perceive as good or problematic to do, in templates that guide the development of policies, or rules that decide who is involved in designing visions for the future of our ageing populations. We chose the empirical ethics approach because it makes it possible to attend to these various ways of thinking about and enacting good and bad ageing. By comparing them and following how they interrelate and shift to the foreground or background, we can understand how some ideas about good and bad ageing come to matter more than others in the European ageing and innovation discourse.

Our case study focuses on the European Innovation Partnership on Active and Healthy Ageing (EIP-AHA), and the creation of a ‘Blueprint on Digital Transformation of Health and Care for the Ageing Society’ (or simply: ‘the Blueprint’), in particular. This Blueprint is meant to act as a vision for the future of (good) ageing in Europe, and as such, to guide policy makers, and also regional, national and EU initiatives for the ageing population. Especially interesting in the Blueprint development is the creation of 12 personas and a series of use case scenarios that depict a number of ‘typical’ old (and also some younger) persons in the context of their everyday life. In this sense, many of the issues discussed in the ageing and innovation literature come together in these personas and scenarios in a tangible form. Our analysis therefore starts from the Blueprint persona ‘Maria’ as an exemplar of European policy-making and investigates how some enactments of good and bad ageing come to matter more than others in the creation of her persona poster and use case scenario, and in the presentation of the problems and solutions that surround her. 

Following this introduction, we provide further details about the theoretical approach and the methods employed. We then introduce our case study, the Blueprint persona Maria, and describe the goods and bads that we find enacted in her persona poster and scenario narrative. In order to understand the diversity, the tensions, and the changes in these goods and bads, we focus on the Blueprint creation process and identify three ‘modes of doing good’ [14] that co-define why Maria and her ageing life are configured the way they are in different policy documents. These modes differ in what they position as important starting points for considering good ageing: (1) so-called ‘key enabling technologies’, (2) the voice of patients, carers and healthcare professionals and (3) a broad understanding of older people that does not reduce ageing to biomedical needs. We discuss the interrelationships and tensions between these visions and analyse how conflicts are dealt with in their concrete enactments in ways that allow some of the goods and bads articulated for Maria to come to matter more, while marginalising others. 

## 2. Theoretical Approach: How Goods and Bads Come to Matter

We make use of a recent body of literature at the intersection of Science and Technology Studies (STS) and Age Studies, which is usually referred to as Socio-gerontechnology [17]. Among other things, this literature has provided rich insights into a range of issues that arise when policy discourses depict the ageing of societies as essentially problematic, with negative stereotypes about older people and later life that come down to impressions of frailty, dependence and, more recently, also health and digital illiteracy [18]. This strand of the literature makes visible how reductionist representations of older people and later life are articulated in a ‘crisis account of ageing’ and then influence and become scripted into the design of telecare systems or robots [12,13]. This often causes problems of acceptance, as such representations contrast with the more positive self-perception of many older adults. Others problematise the focus on ‘user needs and requirements’ that is dominant in many policy discourses and technology design projects, which fails to take into account the values, routines and “little arrangements that matter” [19] (p. 91) to older people in their everyday lives. 

This body of literature tends to explore how policy discourses foreground the problems and challenges in ageing, and it focuses on identifying the ‘bads’ that policy discourses bring about. In contrast, relatively little has been said about the ‘goods’ that are also expressed in these discourses, and how exactly ‘goods’ and ‘bads’ come to matter (or not) in policy responses to ageing. The study by Aceros et al. [11] is an exception. They write that more and more positive approaches to ageing have entered the discursive scene on global, European and regional policy levels over the last couple of years, such as discussions around ‘active and healthy ageing’, ‘independent living’ and ‘ageing in place’. Aceros et al. argue that by articulating ‘good ageing’ in terms of these values, ongoing policy and innovation discourses “create modes of being old that consider certain ways of living better than others” [11] (p. 102). Their work thus illustrates that policy discourses frame ageing in specific and normative ways—not only in terms of the bads to be avoided, but also in terms of the goods to be strived for. 

Our study aims to bring the analysis of goods and bads enacted in the ageing and innovation policy discourse together, and to highlight how they come to matter more or less, and in relation to each other. This seems relevant since the negative depictions of older people indicated by critical age studies indeed inform the ideals of an independent, and both physically and cognitively active, later life as a norm and imperative for a ‘successful’ ageing future [20,21]. The kinds of problems that are made to matter, and the solutions that old age policies propose, are not given, and nor are they independent of each other and the contexts in which they appear. Studying the practices and relationships through which problems and solutions, goods and bads, are shaped, is at the core of what Pols calls ‘empirical ethics’ [16]. In one of the early outlines of this approach, Pols introduced ‘modes of doing good’ to describe not the individual enactments of good care that she found in her studies, but the “pattern of ideals, procedures, routines and knowledge that is oriented towards a specific form of ‘good care’” [14] (p. 320). This idea of ‘modes of doing good’ is also useful for our analysis. We started this paper with the observation that a certain understanding of technological innovations as logical, inevitable and thus inherently good ‘solutions’ for care and ageing, prevails in many old-age policy discourses. This view can be understood as one ‘mode of doing good’, as it comprises several ideals (e.g., promises around AI’s possibilities for personalised health and care), procedures (e.g., the establishment of the EIP-AHA), routines and knowledge (e.g., the selection of health and digitisation experts as informants for the creation of key policy documents). As our analysis will show, however, this is not the only mode of doing good relevant to the ageing and innovation policy discourse and the creation of the Blueprint personas and scenarios. If there is variety in both the ‘modes of doing good’ and in the specific enactments of goods and bads that these entail, then we argue that a relevant question is how some forms of good ageing come to matter more than others (because it will help us to unpack the contingent, co-constitutive making of specific ageing and technology relations as inherently ‘good’). 

It is important to note that drawing on empirical ethics means this paper does not prescribe what good ageing is, or ought to entail, and neither does it merely describe how policy discourses talk about and enact ‘good ageing’. The normativity of the empirical ethics approach lies in its aim to “interfere in the practices studied by opening up implicit notions of good care for (self) reflection” [15] (p. 52). One way to allow such reflection is by comparing different ways of doing good within and across different contexts—what Pols describes as comparative questioning [16]. The study by Aceros et al. mentioned above engages in such comparative questioning. It allows the authors to show that the principles of ‘active ageing’ and ‘ageing in place’ are two goods that are understood in policy discourses as being very much aligned, while in telecare practices, they often appear to be in conflict with each other (for further examples of comparative questioning see [5,22]). Our own analysis engages in comparative questioning in two ways. One is an over-time comparison, where we examine the goods and bads enacted in the 2018 presentation of Maria in her persona poster and follow how they evolve in the 2019 scenario description. In the second form of comparison, we trace connections between the goods and bads articulated in documents about Maria with goods and bads articulated in other contexts of European ageing and innovation policy-making, and most importantly, the EC strategy report on digitising health and care [23]. Together, these two forms of comparative questioning make it possible to better understand how among several ways of thinking about and doing good ageing, some come to matter more than others.

## 3. Methods

This paper engages in a policy discourse analysis. In order to make our case selection, we first collected a broad range of policy documents that provide an overview of ageing policy discourses at EU, global and regional (Basque Country) levels from 2000 until today (see Appendix A). We explicitly searched for ageing policy documents in a broad sense to test the premise that ageing is increasingly framed as part of innovation discourses. This purposive sampling quickly showed that, especially at the EU level, discussions on the future of ageing are indeed very much embedded in economic and innovation policy discourses. The EIP-AHA’s ‘Blueprint on Digital Transformation of Health and Care for the Ageing Society’ is a prime example. It is meant to act as a vision for the future of (good) ageing in Europe, and as such to guide policy-makers, but also regional, national and EU initiatives for the ageing population. 

Especially interesting in the Blueprint development is the creation of 12 personas and a series of use case scenarios that depict a number of ‘typical’ old persons in their everyday life contexts. Many of the issues discussed in the ageing and innovation literature come together in these personas and scenarios in a tangible form. We therefore decided to start our discourse analysis with the Blueprint persona of ‘Maria’ as an exemplar of European policy-making, and to investigate how some enactments of ‘good ageing’ come to matter more than others in this very persona and the scenario descriptions surrounding her, as well as in the processes of creating them. 

These Blueprint personas are intriguing, because, on the one hand, they are enacted almost as real-life individual older persons, but at the same time, they are meant to portray an entire segment of the population. This creates an interesting tension, which is reflected in the way Maria strikes us as a fairly common example of an old age persona. The development of such individualised but typical personas and use case scenarios is increasingly used in policy making, especially in contexts that aim at ‘scaling up’ innovations. By taking such a persona seriously as a manifestation of old age policy-making, we can bring out the underlying discursive forces that configure Maria the way she is and that define which ‘solutions’ are needed in order for Maria to live a good (or better) ageing life. We consider Maria a relevant and highly interesting case for such an analysis since the creation of her persona poster and use case scenario builds on very particular and widespread assumptions about ageing, and also brings to the fore how ageing is indeed configured by many forces outside the realm of old age policy. 

Once we had decided on Maria as the starting point for our analysis of the configuration of ‘good ageing’ in policy discourses, document collection and analysis followed a qualitative explorative approach, where empirical data collection and the specification of concepts informed each other [24]. We gathered a series of documents that help to understand the creation process of the Blueprint of which Maria is a part. First of all, there have been yearly updates since the publication of the first version of the Blueprint at the second European Summit on Digital Innovation for Active and Healthy Ageing in December 2016. These updates provide an overview of the Blueprint partners and the work they have done and will do to achieve the Blueprint goals, including development of the high-impact, scalable scenarios of ‘active and healthy ageing’ that revolve around the creation of 12 Blueprint personas and the identification of what is called key enabling technologies. The Blueprint personas and the Blueprint use case scenarios are thus a second and third relevant series of documents. There are also project-intern documents such as deliverables or recorded webinars, that offer further insights into the Blueprint ‘in the making’. That is, these documents also tell us about why certain decisions were made, which templates were used, or about issues that came up in a discussion and needed to be adjusted. Our iterative process can identify background documents that are not usually used in ageing policy discourse analysis, but which became highly relevant for the focus on discursive practices developed in our research. Together, these different documents formed the basis for analysing how the institutional and historical embedding of the Blueprint into the EC strategy of digitising Europe—including health and care—comes to matter (or not) in the configuration of multiple goods and bads enacted in the creation of Maria and her use case scenario.

These documents (see Appendix B) were coded using AtlasTi. In a first round of open coding, text elements relevant to our research questions were linked to the codes resulting from the concepts. This coding process was complemented by ongoing memo writing, in which new ideas, interpretations and relevant connections or tensions were captured and structured via code and memo groups. We thus identified three ‘modes of doing good’ [14] that seem central to how and why Maria, with her ‘needs’ and ‘solutions’, is framed the way she is within these Blueprint documents. The first mode is related to defining Maria’s ‘unmet needs’ and matching them with digital solutions to respond to these needs. It is in these practices that we can trace how the broader EU strategy of digitising health and care comes to bear on what is proposed as good or better ageing for Maria. The second and third mode of doing good that we identified show that digitisation is not the only starting point for enacting good ageing in the Blueprint, however, a focus on needs and digitisation comes to prevail in the end through the way these other modes are translated into specific practices. In the second round of selective coding, we focused more specifically on these modes and the practices connected to them, in order to identify and understand the dynamics that explain how and why some enactments of the goods and bads in ageing come to matter more than others [24]. 

## 4. Results: A ‘Good’ Ageing Life for Maria

The EIP-AHA is the European Innovation Partnership on Active and Healthy Ageing, launched by the EC in 2011. More than 100 regions throughout the EU form part of the EIP-AHA as reference sites for the deployment and scaling-up of digital innovations for active and healthy ageing. During the Fourth Conference of Partners of the European Innovation Partnership on Active and Healthy Ageing (EIP-AHA) in December 2015, Günther H. Oettinger, then EU Commissioner for Digital Economy and Society, invited participants to work together towards a ‘Blueprint on Digital Transformation of Health and Care for the Ageing Society’. This Blueprint is meant to act as a “common policy vision of European policy makers, civil society, professional organisations and industry on how innovation can transform health and care provision in our ageing society”, and as such, to guide the activities of the EIP-AHA and of its reference sites. In other words, the Blueprint is introduced as a key policy instrument in shaping the future of ageing at the local, regional, national and EU levels. 

This embedding of the Blueprint into a strong EU policy effort for digitising health and care is an important element in the configuration of the forms in which the goods and bads in ageing are articulated in the Blueprint vision. How this embedding matters, however, and the dynamics that can be observed that contest this digitisation-biased prefiguration of ‘good ageing’, are important questions to address. These issues are interwoven and will therefore be addressed in parallel in our results. 

The first section introduces Maria as one of the 12 Blueprint personas. Two key documents are the persona poster for Maria and the 2019 Blueprint update, which includes the high-impact scenario revolving around Maria and her broader ‘care ecosystem’, as it is called. We provide an analysis of the goods and bads that we see articulated in these two documents. The second section takes a closer look into the specific practices and processes of creating the Blueprint personas so as to understand how the embedding of the Blueprint in the EIP-AHA, its actors, funders, protocols and other elements, affect how these personas enact ‘good ageing’. The background documents that tell us about the ‘Blueprint in the making’ are especially relevant. They include the WE4AHA project’s “Deliverable 4.4 Blueprint updates and report on engagement progress”, a recorded webinar and appendixes, such as the call for new Blueprint partners or templates for the persona and scenario development. We identify different practices and modes that help understand the dynamics of how and why Maria is configured the way she is, with all the ‘problems’ and ‘solutions’ articulated as part of her ageing life.

### 4.1. Meeting Maria

**Figure 1:**
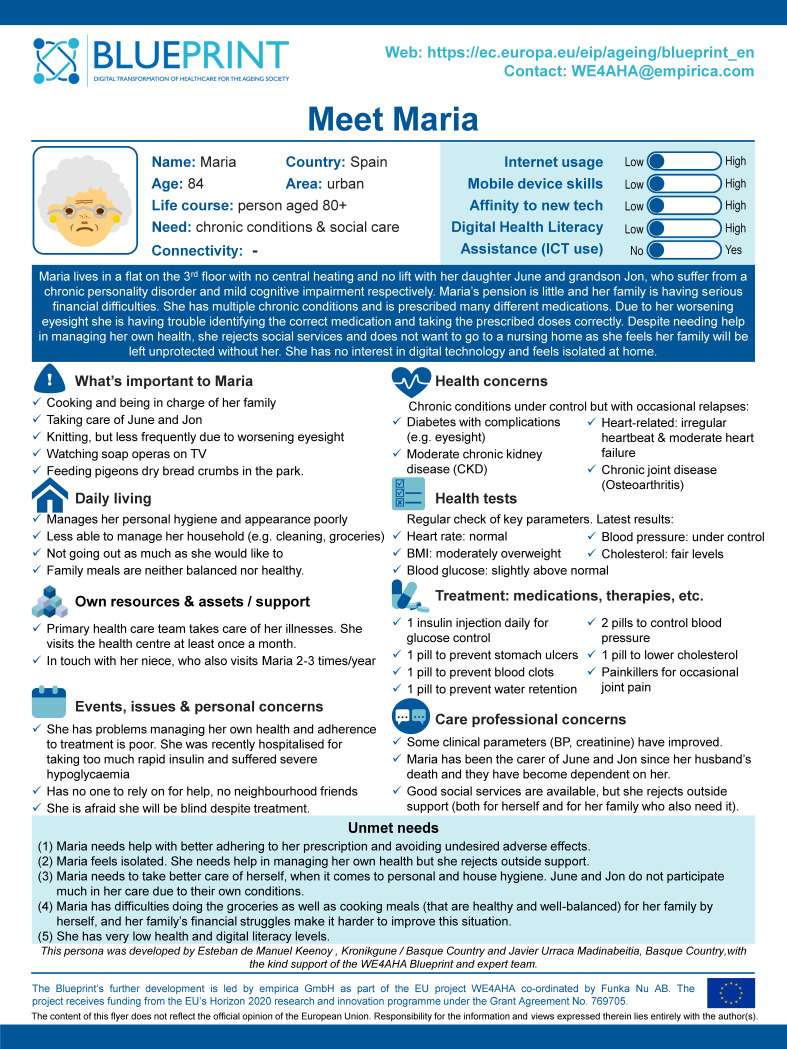
‘Meet Maria’—Blueprint persona poster. The personas originate from the Blueprint stream of work led by Empirica GmbH as part of the WE4AHA project coordinated by Funka NU AB. The project received funding from the EU’s Horizon 2020 research and innovation programme.

One important part of the Blueprint development process is the creation of a set of Blueprint personas, presented in 12 persona posters. Who are they? One is named Maria, an 84-year-old lady living in an urban area in Spain together with her daughter June and her grandson Jon. Maria suffers from several chronic conditions and it is difficult for her to always find the right medication. She needs assistance but often refuses help as she is afraid to be taken to a nursing home. The introductory paragraph about Maria (see Figure 1) ends with the remark that “(s)he has no interest in digital technology and feels isolated at home”. 

Which goods and bads do we find articulated in Maria’s persona poster? Whose perspective is presented in these goods and bads? One perspective is that of Maria herself. The activities listed as ‘What’s important to Maria’ describe routines that Maria enjoys, practices that for her are part of a ‘good ageing life’. They include cooking and taking care of June and Jon, knitting, watching TV or feeding pigeons with dry breadcrumbs in the park. Other goods and bads from Maria’s perspective are left more implicit, and described in what Maria worries about, what she is afraid of and what she actively seeks to happen or to avoid. One example is her worry about being taken into a nursing home (i.e., a ‘bad’ that Maria actively seeks to avoid) interlinked with her wish (i.e., a good Maria strives for) to age at home in order to take care of her daughter June and grandson Jon: “… she rejects social services and does not want to go to a nursing home as she feels her family will be unprotected without her”.

A central aim behind the development of the Blueprint personas is to move beyond a mere biological understanding of ageing and also consider the everyday routines and interactions of older people in relation to their wider social environment [25]. In addition to the perspective of Maria herself, the persona poster therefore also tells us about the concerns of the (fictitious) care professionals who interact with Maria on a regular basis (see bottom right of Figure 1). Maria’s clinical parameters for blood pressure (BP) and creatinine have improved—good news. By contrast, the fact that Maria does not accept the support of social services is articulated as a ‘bad’ from the perspective of the care professionals, since, in their view, Maria needs support. The ‘good’ they strive for is a situation where Maria, as well as June and Jon, make use of the available social services to alleviate the care burden they experience.

The headings “What’s important to Maria” and “Care professional concerns” explicitly indicate to the reader whose perspectives are expressed in these parts of the persona poster. In other parts of the poster, this remains more implicit. At the top right of the persona poster, for instance, a visual presentation of Maria’s digital skills attracts the reader’s attention. The graph underlines that Maria does not use any kind of (assistive) ICT devices and nor does she have the skills to use them. What kinds of goods or bads are expressed here? From whose perspective? Maria herself “has no interest in digital technology”. Seen from her perspective, low levels of internet usage, mobile device skills or affinity to new technologies are thus no big surprise and logically nothing that Maria herself would consider an issue that needs changing. Yet, her low digital literacy is listed as an ‘unmet need’ at the bottom of the persona poster. Each point in the list of ‘unmet needs’ describes one aspect of Maria’s life as a ‘bad’ that should be addressed and avoided, and—sometimes explicitly, sometimes implicitly—suggests a better or good situation to work towards. As our analysis will show, the summary of unmet needs is the part of the persona poster that most strongly informs the ‘solutions’ proposed to Maria but also wider policy and health innovation activities. It is therefore both interesting and important to realize that the goods and bads enacted in the persona poster express different and sometimes conflicting perspectives and interests, and to unpack how they came to matter in the making of the Blueprint personas. 

One year after the creation of Maria and the publication of her persona poster, the Blueprint partners released the 2019 Blueprint update, an official report which includes the first examples of the Blueprint use case scenarios. These scenarios describe, analyse and ‘solve’ incidents happening in the life and care environment of the Blueprint personas. For Maria, this is an incident where the GP found Maria in her home in a weak state (hypoglycaemia), partly due to the wrong medication given by her worried daughter June. After a short description of this event, the second section of the scenario text suggests a number of factors that explain why the event happened:

“Maria has a basic education level, although she is able to read and write. Maria needs to take better care of herself, when it comes to personal and house hygiene. Her daughter and grandchild do not participate much in her care due to their own circumstances.

The main factor that has contributed to this serious episode of hypoglycaemia is undoubtedly the lack of knowledge of the caregiver, Maria’s daughter. She has assumed responsibility for caring for her mother without adequate knowledge and possibly without psychological stability, which has led her to misinterpret the situation and act in an unreflective way. Neither she, nor Maria, understand how to administer Insulin correctly or how to use a glucometer. Putting the handling of rapid insulin in June’s hands may have not been wise and, without the person having appropriate training, can be considered a risk factor. The endocrinologist had not been aware of the psychosocial conditions of Maria and her daughter. Given that situation and the age of the patient, the general practitioner would have proposed the setting of less exhausting control goals for Maria’s care plan. Both communication and coordination were inadequate.”

[25] (p. 14)

The lack of knowledge that both Maria and her daughter June have about Maria’s health and about which medications to take when and how is identified as the main cause of the event, and it is concluded that the underlying issue is one of insufficient communication and coordination in Maria’s “care ecosystem” (as the Blueprint partners call it). We find the first point, Maria’s low health literacy, already on the list of unmet needs in the persona poster. Statements about insufficient communication and coordination among those involved in Maria’s care articulate another ‘bad’ that prominently figures in health and ageing policy and innovation discourses [26]. The scenario text goes on to further explain the event as also being caused by Maria’s wishes and fears, and her broader socio-economic situation:

“There has not been a care network established around Maria. Maria feels isolated. She has difficulty in doing daily shopping and cooking meals for the family. The meals she prepares are not really healthy. In addition, she faces financial difficulties which do not improve the situation. Her niece does not take part in any decision making about health or social care. A social worker has visited Maria twice but she has rejected help. She is afraid that she will be taken to a nursing home and her daughter and grandchild institutionalized. She is alone for most of the day and does not want to leave her house to go to a nursing home, but clearly she needs help. Home support with cleaning, shopping and cooking would greatly improve Maria’s condition.”

[25] (p. 14)

While the persona poster still more or less explicitly distinguishes between the perspective of Maria and of her care professionals, this scenario extract brings them together in one narrative. What we see is a gradual alignment of goods and bads through a blurring of different and sometimes diverging perspectives. 

After this explanation of the causes of the event, the scenario text continues with a section analysing the key actors involved, and their (potential) roles in improving the overall situation of Maria. In addition to Maria, her daughter and a niece living further away, these key actors include the primary health care team and the local social services. In a subsequent fourth section of the scenario, specific propositions are made regarding how to deal with and prevent similar incidents in Maria’s life. This section four of the scenario brings together those problems identified as causing Maria’s incident with suggestions about ‘solutions’ that would enable Maria to live a better ageing life:

“All the key actors, including Maria, her daughter, her niece, home support and healthcare professionals, need to exchange secure information among themselves. They need to coordinate their goals, actions and avoid redundant and potentially harmful interventions. An information system that collects relevant data and information to enable monitoring of care plan related activity status and progress is needed, i.e., information to support the exchange of glucose level data and information about symptoms would be helpful. Alerts could be handled by the eHealth 24/7 centre, primary care and/or emergency services.

It would be necessary to make sure that all the key actors understand correctly the relevance of proper diet habits, handling insulin and using a glucometer. A health education program, customized to Maria’s basic literacy skills and June’s conditions, should be implemented via a Patient Empowerment Platform. The aim is to recommend, implement and have all the actors adhere to Maria’s personalized treatment goals and interventions…”

[25] (p. 15)

An exchange of information between everyone involved in Maria’s care ecosystem, together with the better coordination of everyone’s goals and actions are the two core solutions proposed. An additional ‘good’ the scenario authors strive for is improving the health literacy of Maria and her daughter. These three goods together exactly align with what, in Section Two of the scenario, had been foregrounded as the major causes behind Maria’s incidence of hypoglycaemia, as bads that require attention. The scenario description then provides further explanations about how this exchange of information, better coordination and the increased health knowledge of Maria and her daughter can be achieved. The close cooperation of all key actors, the scenario suggests, can be realised through the use of interconnected ICT tools and services—such as an electronic health record, a personalised care development platform, a wearable skin glucose monitoring device and a patient education platform—which continuously communicate and transmit remote monitoring signals. Maria’s subsequent ‘good ageing life’ is illustrated in the following retrospective narrative in the 2019 Blueprint update:

“Recently, her niece has been increasingly involved in helping Maria to maintain a healthy diet and helping her with the new ICT tools that Maria has started to use. Maria’s niece photographs her daily meals, which are analysed by her nutritionist—part of a multidisciplinary nursing team responsible for her health—for nutrient content and quantities. A new digital wristband tracks Maria’s physical activity, glucose and insulin levels, determines the timing and dosage of her insulin intake, and transmits the information to her niece, who is able to make informed decisions for Maria. She also helps Maria to understand her health on a simple level. The data collected by the portable monitoring device is transmitted to a local 24/7 call centre, which evaluates Maria’s values when critical observations are made and thus provides her multidisciplinary nursing team with important decision support. In difficult situations, a member of the nursing team can either immediately give instructions to the niece or advise Maria at her next appointment.

It is a completely new experience for Maria to learn how to get health, lifestyle and treatment advice from a patient empowerment platform. She does not really understand the medical background, but her niece has told her that she could trust the information on the platform because it comes from the joint results gathered by her multidisciplinary care team, who all access her personal data and set new recommendations and treatment goals for her for the benefit of her health. She gets access to the platform through a tablet designed specifically for older patients. Through this platform, a health education programme tailored to the (health) literacy skills of Maria and her daughter improves the understanding of the relevance of having correct eating habits, and insulin and glucometer use.”

[25] (p. 19)

In summary, through the Blueprint persona poster we have got to know Maria as an 84-year-old Spanish lady who faces a range of difficulties in her everyday life, but who also has certain things that she likes doing and that are important to her, and other things that she is afraid of. Other people also worry about Maria’s health and social situation and think about ways to allow her to live a good (or better) ageing life. The persona poster combines these perspectives and articulates a variety of different and sometimes clashing goods and bads. From this variety of goods and bads, the Blueprint use case scenario narrative identifies the low health literacy of Maria and her daughter June, together with the insufficient communication and coordination in Maria’s broader care ecosystem, as major challenges that need to be addressed in order to avoid unwanted incidents, and to allow Maria to live a better ageing life. The close cooperation of all actors available in Maria’s care and daily life, enabled through the use of interconnected ICT tools and services that constantly collect and share data, is described as the ‘solution’ to these challenges. The questions arising are: How do the persona and scenario authors come from an identification of rather mundane problems in Maria’s life to a solution that is based on the constant use of very sophisticated technologies? For whom is this ‘solution’ a solution, especially as Maria is introduced with the remark “(s)he has no interest in digital technology and feels isolated at home” [27] (p. 1)? 

The next section examines the ‘Blueprint in the making’ to address the above questions. In particular, we trace three ‘modes of doing good’ [14], which—through their specific pattern of knowing, talking about and dealing with ageing—co-define why those problems and solutions, identified in the persona poster and scenario, are articulated in the first place. The first mode considers good ageing primarily from the technological innovation perspective. The second, in contrast, positions patients, carers and healthcare professionals as the most valid starting point for a health and ageing policy. The third mode emphasises that ageing is more than a biomedical process, and that it is therefore not only the (health) needs of older people which should be taken into account, but also their values, aspirations and everyday interactions with their broader socio-material environment. In our analysis, we pay specific attention to the practices through which these three modes of doing good inform the kinds of goods and bads that come to matter in the creation of the Blueprint persona posters and scenarios, and which ones thereby come to matter more. 

### 4.2. Mode One: ‘Good Ageing’ and Key Enabling Technologies

The WE4AHA Blueprint project Deliverable 4.4 describes the different steps in the persona and scenario creation process. It is not a sequence of practices following one after the other, but rather a set of several parallel and interwoven activities. A core part of the scenario development is what the deliverable describes as “Matching persona needs and their typical actions with possible solutions, services, technologies” [28] (p. 24). As the term ‘matching’ also indicates, one of the Blueprint activities was to define a pool of “possible solutions, services, technologies” from which the scenario authors then had to select those that best fit the identified ‘unmet persona needs’ and the persona’s ‘typical actions’. For Maria, this included the online health education programme which ‘matches’ her need to better know which medications to take when and how. Defining this pool of potential solutions, often only referred to as KETs (Key Enabling Technologies), however, was a process that started before the persona creation. If the delineation of possible solutions started before Maria as a Blueprint person was even created, then what did inform the decisions about what to include within this pool?

One of the first activities in the Blueprint development was to define four key topic areas that would guide the Blueprint activities: (1) ‘Data analytics for predictive risk stratification and prevention’, (2) ‘Proactive prevention through empowerment, self-management, monitoring and coaching’, (3) ‘Digital solutions for connected health’ and (4) ‘Digital support for integrated care’. As the deliverable document explains:

“Four initial key topic areas have been chosen that were identified jointly by EU actors on both the demand and supply sides prior to the first Blueprint Call for Engagement under WE4AHA. *These four areas were selected to represent digital health and care priorities* and have undergone various cycles of discussions and reviews.”

[28] (p. 10, italics added)

The digital health and care priorities represented by the four topic areas are formulated in the EC Communication on the Digital Transformation of Health and Care, 2018. They are summarised as:

- “Citizens’ secure access to and sharing of health data.
- Better data to advance research, disease prevention and personalised health and care.
- Digital tools for citizen empowerment and person-centred care.” [23] (p. 3).

Looking back on both the unmet needs and solutions articulated in Maria’s persona poster and scenario, it can be seen that there is not only a match between these unmet needs and solutions, but then a further match with the four Blueprint key topic areas that represent the EC priorities. Improving Maria’s health literacy by means of an online patient empowerment platform fulfils the EC priority to implement ‘digital tools for citizen empowerment and person-centred care’. In other words, these EC priorities co-define what ends up being proposed as ‘good ageing’ to Maria. When good ageing is enacted as a matter of avoiding health problems and harmful interventions through data collection and sharing in the scenario for Maria, or as a matter of improving Maria’s health education via patient empowerment platforms, then we can say that good ageing for Maria is enacted in relation to economic or digitisation policy. The note that Maria herself has no interest in digital technology further emphasises this point.

Our attention to the Blueprint in the making has revealed the discursive and material traces through which the EC strategy for realising the digital single market pre-frames the possibilities for different goods to matter (more) in Maria’s life. This, however, is in no way an evident, clear, or a static result. Although the blueprint documents make it very clear that one of the goals is to “identify and specify key ICT enabling technologies and high-impact user scenarios in AHA” [28] (p. 22), realising digitisation is not the only ‘mode of doing good’ or starting point for thinking about and working towards ‘good ageing’ that we can find in the Blueprint. Importantly, it is also not the only consequence, as ageing is also constituted along the way. In the next section, we will therefore specifically look at two other modes of doing good that contest this digitisation-biased prefiguration of ‘good ageing’. 

### 4.3. Mode Two: ‘Good Ageing’ and Attending to Patients, Carers and Healthcare Professionals

The first published version of the Blueprint clearly states that “patients, carers and healthcare professionals should be placed at the centre of healthcare innovation and policy reforms” [29] (p. 4). We analyse such statements as a second mode of doing good that gives direction to the Blueprint development process. What are the specific practices through which this emphasis on putting patients, carers and healthcare professionals centre-stage is enacted? One way of engaging with this question is to see who, from the ‘demand side’, was invited to contribute as a partner to the Blueprint development. The core group working on the Blueprint, the so-called Blueprint champions (see [29] (p. 29) for the full list of champions), was established from within organisations already working closely together in the EU’s active and healthy ageing and innovation domain. In 2017, WE4AHA launched a call to add to this core group what they specified as ‘demand side representatives’:

“The aim of this call is to enlarge the group of contributors to the Blueprint’s further development and strengthen the demand side representation. We seek to activate participation of demand side representatives who are investing in or implementing digital health and care solutions at scale between now and end of 2018.”

[28] (p. 67)

This call extract indicates how the boundaries of ‘demand-side participation’ are drawn. The deliverable lists some examples of relevant groups of demand-side representatives: regional authorities, health and care providers. Within these groups of relevant actors, however, the selection is further specified to include only those “who are investing in or implementing digital health and care solutions at scale between now and end of 2018” [28] (p. 11). This specification is translated into two clear selection criteria:

- “Relevant expertise of the candidate matching the topic area.
- Relevance of the digital health solutions, which the region/organisation is investing in or has implemented at scale, to the selected topic area”. [28] (p. 11)

In the selection (and constitutive exclusion) of who is to represent the ‘demand side’ in the development of a vision for the future of ageing and care in Europe, we thus can observe practices through which a certain expertise and experience with digitisation of health and care (note: not ageing and care) comes to count as meaningful. In other words, the recurring statement about putting patients, carers and healthcare professionals centre stage and involving them as active contributors in the development of the Blueprint is a form of configuring ‘good ageing’ that has the potential to go beyond, and in some way challenge the influence of the Blueprint’s contextual embedding, however, as the example of this Blueprint call and its selection criteria shows, the way in which the idea of demand-side representation is enacted in the Blueprint is in fact again pre-framed in terms of digitisation. Against the contextual embedding of the Blueprint, this is not an astonishing result, but the analysis helps to understand and illustrate how, in a very specific sense, the background of the Blueprint has discursive but also material effects (e.g., the constellation of partners) for the ongoing configuration of ageing in the Blueprint discourse.

### 4.4. Mode Three: ‘Good Ageing’ and Values, Interactions and Complex Human Lives

A similar dynamic of first articulating what is important in a broad way and then limiting possibilities through further specification can also be seen in relation to a third mode of doing good: those recurring statements and practices that revolve around the ideal of seeing older people and their ageing lives as something more than a biological body with health needs. As argued in the latest Blueprint update, part of the aim of putting patients, carers, healthcare professionals and their interactions centre-stage is to acknowledge and better understand “the richness and diversity of complex health and care ecosystems” [25] (p. 5). We learn not only about Maria’s body and her diseases, from the persona description of Maria and the scenario of her experiencing an acute episode of hypoglycaemia, but also about the environment in which she lives, the people she interacts with, her financial difficulties, her fears and the daily routines that bring joy to her life. It is through the third mode of doing good that everyday routines such as Maria’s cooking for her daughter and grandson, as well as what she values and fears, come to count as meaningful elements to consider:

“While traditional persona profiles do not recognise psychological or psycho-social forces within people and their health care choices and outcomes, research has shown that ‘failing to recognise such cognitive and behavioural patterns of perception and action can affect both short-term and long-term success with interventions directed toward managing a disease or adopting wellness’. The Blueprint work on personas therefore took behavioural characteristics into consideration by including descriptions such as people’s trust (or lack of) in care professionals, their self-management capabilities, and specific details on their character (e.g., prone to aggressive behaviour or tendencies to reject outside support), among others.”

[28] (p. 22)

Interestingly, the creation of personas was motivated in the first place by the idea that it would enable a ‘deeper understanding’ of older people. In this way, an effort to go beyond traditional approaches, that tend to employ a mere biomedical understanding of ageing and do not take into account the broader environment, routines, or values, is made to matter in some statements. Such effort also materialises in the detailed and socially rich description of Maria in her persona poster. When we look closely at how this intention is specified in further discursive and material enactments, however, we note that this effort has already disappeared in the next sentence of the same document:

“The creation of personas is one of the proven successful concepts that enable companies to understand their potential users more, by considering their needs, aspirations, attitudes, dreams, and other relevant characteristics. Within the context of the Blueprint work and objectives, personas were developed to envision realistic health and care needs of certain groups in the society.”

[28] (p. 26)

While the potential contribution of technologies towards ‘good ageing’ is enacted in the first sentence as a matter of understanding and attending to ‘needs, aspirations, attitudes, dreams and other relevant characteristics’, the next sentence reduces this back to ‘realistic health and care needs’. Such dynamism between going beyond needs but then quickly falling back into a focus on realistic and unmet needs goes together with another common dynamism where the individualistic idea of a user is distilled from a socially rich description of the persona’s life [30]. Both are recurring observations in our analysis. During a WE4AHA webinar on the 12 Blueprint personas, held in 2018 and made public on YouTube, the presenter of persona ‘Randolph’ first introduced all the details about Randolph, his challenges, wishes and everyday joys, and then concluded that the technology and healthcare service providers who will eventually use this persona poster, should in the first place respond to the identified “unmet needs”: “So really, for Randolph, these [the unmet needs] are the kind of four really key areas that may be for solution providers to actually consider and think about” (minute 36:24). This message is also emphasised in the design of the persona poster by visually foregrounding Randolph’s unmet needs [31]. We can thus argue that the dynamics described in this section—where details ‘beyond needs’ are articulated as meaningful, but then come to matter less because ‘unmet needs’ with a predominant focus on health issues are emphasised instead—arise not only in the writing of Blueprint documents, but also materialise in the persona poster’s visual form (and possibly also in the practices of the addressed solution providers). 

In summary, our analysis of the Blueprint creation process has revealed three modes of doing good that, we argue, can help us understand why Maria and the problems and solutions presented as part of her ageing life are configured as such in the persona poster and the use case scenario. What has also become clear, however, is that the ideals behind these three modes of doing good can be in conflict with each other. This is the case when, for instance, Modes Two and Three propose putting Maria centre stage with her values and fears, and thus taking her disinterest in digital technology seriously, while Mode Three is all about helping Maria live a better life through the deployment of new technologies. The next section will focus on such conflicting situations between the modes of doing good in Blueprint development in order to discuss how the ways in which such tensions are solved affect the kinds of goods and bads that are marginalised, and which ones come to matter more. 

## 5. Discussion: Dynamics of Mattering and Mattering More

We have identified a number of practices from our empirical material that illustrate how the contextual embedding of EU ageing and innovation policy into the broader EU digitisation strategy becomes constitutive of what is to be strived for (i.e., ‘goods’) and what is to be avoided (i.e., ‘bads’) when living as an older person. In Maria’s case, a health education platform and information sharing between Maria’s health and social care providers are solutions—or ‘goods’—that not only ‘match’ what is specified as Maria’s unmet needs, but as we have shown, also ‘match’ the three priorities of the EC strategy to digitise health and care. Such a seemingly straightforward alignment between the wishes and needs of older people and the EC’s aim to foster gerontechnological innovation is common in discourses in the active and healthy ageing domain. In his study of the development and test use of a telecare monitoring system, Neven [1] describes how the idea that older people want to age in their own home independently is positioned as ‘evidently the right thing to do’, and gerontechnological innovations as the obvious means of enabling this. Many older adults indeed express a wish to age in their known environment, which is also portrayed in Maria’s persona poster. When an online health education platform and data sharing are proposed as solutions to Maria, these solutions, or ‘goods’, thus, do not necessarily clash with Maria’s own ideas of how she wants to age. Using an online health education platform and data sharing is, however, only one way of realising Maria’s wish to stay at home. In fact, this way of enabling her to age in place becomes less obvious as ‘the right thing to do’ when we take into account that Maria “has no interest in digital technology and feels isolated at home” [27] (p. 1). This example demonstrates a crucial point that empirical ethicists try to convey: “an ideal may inspire different practices, even if the overall ideal is said to be the same” [15] (p. 55). In other words, ageing-in-place can be realised in many ways. None of them are self-evident, but an outcome of the interactions of diverse elements. One dominant element in our example is the European digitisation agenda. This leads us to question the extent to which the forms of ‘good ageing’ proposed in the Blueprint vision really arise from caring about the needs and values of Maria, let alone from caring about those of the older people Maria is meant to represent. Is this caring at all separate from the political and economic goal of digitising European health and ageing?

What we found in the Blueprint documents defies simple answers to this question. On the one hand, there is a clear interrelationship between what is proposed as good ageing in Maria’s scenario and what is proposed as good ageing in EC policy priorities. This interrelationship also stretches into the kinds of ‘bads’ that become foregrounded in the unmet needs. In line with Willem’s concept of *varieties of goodness* [5], however, we also identified other forms of enacting good and bad ageing that are not prefigured by the European digitisation agenda. These revolve around the two other modes of doing good in the Blueprint development—the idea that “patients, carers and healthcare professionals should be placed at the centre of healthcare innovation and policy reforms” [29] (p. 4) and the argument that older people should not be reduced to their bodies and health needs, but that their “aspirations, attitudes, dreams, and other relevant characteristics” [28] (p. 26) also deserve attention. In contrast to the former, these two modes enact older people—embedded in a rich socio-material environment—as relevant, valid and eligible starting points for healthcare innovation and policy reform. It is notable that these ideas do not exist only as side remarks, but recur throughout several documents, materialise in graphs [32] (p. 21), in worries about becoming blind despite treatment and in lists about “What’s important to Maria” [27] (p. 1). In fact, they were the basis of creating the Blueprint persona posters in the first place [28]. It thus seems important to take them seriously as part of the Blueprint’s practices of articulating and enacting goods and bads in ageing. To come back to our question about who or what is being cared for in the Blueprint’s vision, we can thus draw a first conclusion: taking into account all the different Blueprint documents, the variety of goods and bads enacted in relation to Maria not only arise from caring about a political and economic goal of digitising European health and ageing (first mode), they also arise from attention to older people as complex social beings with attitudes, dreams and fears, embedded in a broader socio-material web of interactions (second and third modes).

There is, however, yet another aspect we need to discuss in this regard. Although we found this variety of goods and bads in the Blueprint documents, this does not yet say anything about which ones actually come to matter, and how. Indeed, when we take a cross-context and over time perspective, we realise that the different modes of doing good, and thus also the different goods and bads enacted with them, do not come to matter equally. Something shifts in the way Maria and her ageing life are configured in the persona poster, compared to how they are configured in the later use case scenario. In Maria’s persona poster, bio-medical concerns and her ICT skills are included as relevant elements to consider, but more mundane everyday details are also included, such as the list of routine activities she enjoys doing. The three modes of doing good thus all inform the kinds of goods and bads enacted in the persona poster, yet when looking at the solutions that are proposed for Maria in the scenario narrative, most of the more mundane aspects no longer seem to play a role. The scenario sketches a ‘good ageing life’ for Maria that has ‘unmet needs’ and highly sophisticated digital technologies to respond to these needs at its centre. That is, the goods and bads in Maria’s persona poster that arise from an attention to ageing as something broader than needs are either excluded or come to matter less in the use case scenario. What comes to *matter more* are those goods and bads that are part of practices which foreground ageing as a matter of ‘unmet needs’ and digitisation as the obvious means to solve these needs. 

How can we, from an empirical ethics of care perspective, make sense of these dynamics of mattering and mattering more? One answer lies in looking closely at the ways in which tensions between the different modes of doing good are solved. In the example above, we argued that the mismatch between Maria’s disinterest in digital technologies and the sophisticated technologies proposed in her scenario description is a result of matching the ‘problems’ in Maria’s life with ‘solutions’ pre-framed in the context of EC priorities. Practices of defining what counts as ‘unmet needs’ are key to this matching process. Staging Maria’s low health and digital literacy levels as unmet needs means that a connection to online health education and patient empowerment programmes is established. At the same time, the remark about Maria’s disinterest in digital technologies is marginalised and left forgotten. Defining problems and matching them with solutions is thus a first type of practice that explains how some goods and bads come to matter more than others.

Another way of dealing with conflicts between modes of doing good is to first articulate ideals in a broad way (where conflicting notions of the good can exist alongside each other), and then gradually and partly align conflicting goods through further specification in enactment. Regarding the second mode of doing good, we showed how involving patients, carers and healthcare professionals as central actors in the Blueprint creation process is expressed as ideal in the Blueprint documents. This broad framing leaves room for this ideal to be in conflict with the first mode of doing good, where good ageing is considered from digitisation as the central starting point. The specific practices of selecting demand-side representatives in the 2017 Blueprint call, however, enact this ideal in such a way that only those with previous experience and expertise in digitisation are eligible. By way of specifying demand-side representation, the ideal of having patients, carers and health-care professionals centre-stage is thus brought into alignment with the digitisation focus of the first mode of doing good. Similar observations can be made regarding the tensions between Mode Three and Mode One. In an explicit effort to avoid reductionist representations of ageing and attend to the values, fears and everyday events important to older people (Mode Three), the Blueprint partners have included a socially rich description of Maria and her everyday life in interaction with other people, materials and places. Our empirical analysis of background documents such as the WE4AHA project Deliverable 4.4, however, has shown that while the potential contribution of technologies towards good ageing is enacted in some sentences and practices as a matter of attending to ‘needs, aspirations, attitudes, dreams and other relevant characteristics’, the next sentence reduces this back to ‘realistic health and care needs’ [28] (p. 26). This move from a rich description of Maria’s lived reality to a focus on unmet needs—which reappears in the visual and oral foregrounding of ‘unmet needs’ in the persona poster and the Blueprint webinar—is a form of specification that aligns the third mode of doing good with the first mode. On the one hand, ageing is understood in a broad way, where including details such as Maria’s cooking and what she values and fears come to count as meaningful elements to consider. On the other hand, this understanding of ageing is specified and enacted in such a way that what we are really expected to focus on are certain ‘unmet needs’, and everyday routines or values shift to the background.

In summary, we identified two types of practices that help explain how and why—despite the variety of goods and bads identified—a focus on ‘unmet needs’ and sophisticated digital solutions come to matter more in Maria’s use case scenario—and thus also in the European vision for the future of health and care in an ageing society. Defining unmet needs in Maria’s life so that they match solutions envisioned in the broader EU strategy for digitising health and care is the first type of practice. Specifying alternative ideals so that that their enactment aligns them with the dominant ideal is the second. It is important to remember that these dynamics of mattering and mattering more are ongoing and open-ended. By making these practices explicit, we take away some of the self-evidence attached to both ‘problems’ and ‘solutions’. The aim is not to prescribe one version of good ageing or good care, but to invite and engage in processes of trying, experimenting and tinkering with multiple and sometimes conflicting goods and bads in relation to the situation at hand [15,22].

## 6. Conclusions

This paper has provided an empirical analysis of goods and bads enacted in the European policy vision for the digitisation of health and care in ageing societies. We set out to answer two questions that figure as important but open issues in debates around (good) ageing and the role of technology therein: (a) What can we—based on empirical insights—say about the practices and processes through which the increasing dominance of technology in ageing is made possible in policy discourses? (b) What role do these practices and processes play (or do not play) in the configuration of how some ideas about good and bad ageing come to matter more in these discourses? 

Drawing on the Blueprint persona of Maria as a common example of European old age policy-making, we analysed a variety of practices that render visible how the idea of technology and data sharing as evidently the right path towards futures of (good) ageing—a point that many scholars problematise [2]—comes to prevail in these discourses. In fact, it is not merely an idea but a first ‘mode of doing good’ that actively shapes the Blueprint development. It comes back in ideals, knowledge, routines and procedures, such as the selection process regarding who is invited to represent the ‘demand side’ in the Blueprint creation. Our study thus adds a more nuanced picture to existing studies of the ageing and innovation discourse, of the specific ways through which the increasing and seemingly inevitable incorporation of technology into ageing is accomplished. It shows the practices through which ageing is understood, talked about and enacted primarily in terms of ‘unmet needs’ that (are made to) match a European digitisation agenda.

Our empirical material leads us to argue that presenting ageing and innovation policy discourses as reducing older people to individualised users with biomedical needs (e.g., [30]), and as considering good ageing primarily in terms of digitisation, does not do justice to these discourses either. If we look closely at the discourse documents, we see that Maria is not only framed negatively. Attending to Maria as someone who has a history, who has dreams and fears and who lives in interaction with other people, places, animals, medications and other materials, demonstrates that other ‘modes of doing good’ are also relevant in the Blueprint creation. The ways in which goods and bads in ageing are imagined and enacted in the Blueprint discourse is more diverse than studies of the ageing and innovation discourse would suggest. 

Yet again, although we observed three different modes of doing good in the Blueprint documents, what is proposed at the end of the scenario narrative as ‘good ageing’ for Maria is in the first place an effect of the political and economic goal of digitising European health and ageing. Constant data sharing, health education platforms and personal care plans may fulfil Maria’s wish to age at home, but they also ignore her disinterest in digital technology. Studying how such tensions appear in the persona poster but disappear in the use case scenario shows that dominant enactments of ageing as certain unmet needs, together with digital solutions to serve these needs, are not inevitable, but the results of discernible practices that can be carried out differently. Drawing on the empirical ethics of care literature, while also contributing a new attention to policy discourses as sites where goods and bads are being shaped, we thus provide a form of critique for ageing and innovation discourses that not only unmasks the often ageist stereotypes entrenched in them, but that also brings out the political, social and material forces that enact and keep such stereotypes in place—a critical mandate that is crucial for social sciences and humanities studies of ageing and technology [33]. This then provides inroads for thinking about alternatives to these images, and the practices that can enact them. We hope that the insights of our study help to break out of the rut of seeing digitisation as ‘inevitably the right thing to do’. As we have shown, the first signs of this are already there. It is now necessary to also allow those enactments of good ageing that start from caring (in Puig de la Bellacasa’s [34] sense of ‘matters of care’) about older people and experiences of later life, to come to matter more. 

## Figures and Tables

**Figure A1:**
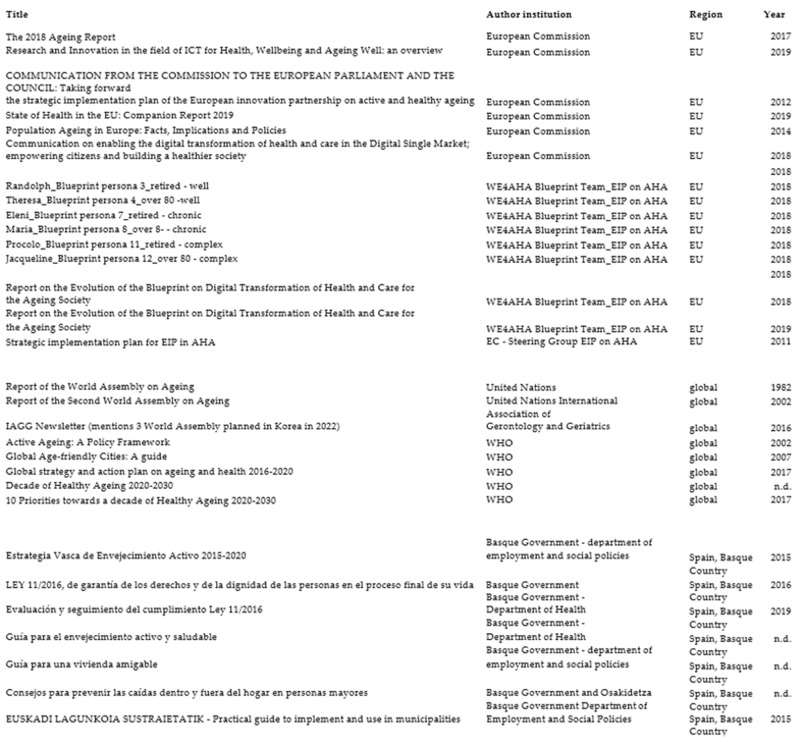
First round of ageing policy discourse document collection.

**Figure A2:**
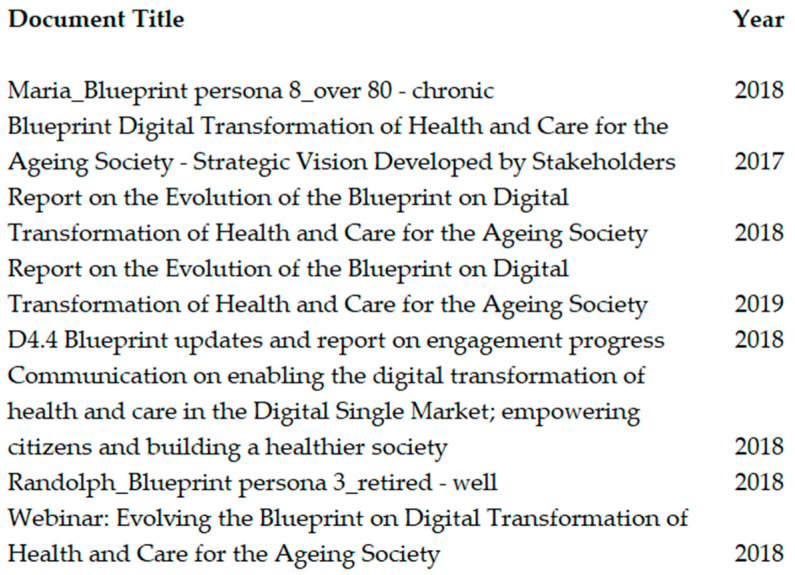
Coded documents.

## Data Availability

The data is stored safely on an encrypted Utrecht University SurfDrive account. It is available upon request and will be made accessible through the data storage system of the Copernicus Institute of Sustainable Development (currently being developed).
